# Impact of the Freeze-Drying Conditions Applied to Obtain an Orange Snack on Energy Consumption

**DOI:** 10.3390/foods10112756

**Published:** 2021-11-10

**Authors:** Marilú Andrea Silva-Espinoza, María del Mar Camacho, Javier Martínez-Monzó, Nuria Martínez-Navarrete

**Affiliations:** Food Investigation and Innovation Group, Food Technology Department, Universitat Politècnica de València, Camino de Vera s/n, 46022 Valencia, Spain; masiles@doctor.upv.es (M.A.S.-E.); mdmcamvi@tal.upv.es (M.d.M.C.); xmartine@tal.upv.es (J.M.-M.)

**Keywords:** orange puree snack, shelf temperature, chamber pressure, primary drying, secondary drying, energy consumption

## Abstract

Nowadays, the consumer is looking for healthier, more attractive, ready-to-eat, and safer foodstuffs than fresh products. Despite freeze drying being known for providing high added value products, it is a slow process which is conducted at low pressures, so, in terms of energy consumption, it turns out to be quite costly for the food industry. With the purpose of obtaining a freeze-dried orange puree, previously formulated with gum Arabic and bamboo fiber, which can be offered to consumers as a snack at a low economic cost, the impact of the process conditions on energy consumption has been considered. The product temperature evolution and the energy consumption were registered during the drying of frozen samples at different combinations of chamber pressures (5 and 100 Pa) and shelf temperatures (30, 40 and 50 °C). In each case, the time processing was adapted in order to obtain a product with a water content under 5 g water/100 g product. In this study, the secondary drying stage was considered to start when the product reached the shelf temperature and both the pressure and the temperature affected the duration of primary and secondary drying stages. The results obtained led to the conclusion that the shorter duration of the process when working at 50 °C results in significant energy saving. Working at a lower pressure also contributes to a shortening of the drying time, thus reducing the energy consumption: the lower the temperature, the more marked the effect of the pressure.

## 1. Introduction

In recent years, the incentive to consume greater quantities of fruit has been promoted by both governmental and non-governmental organizations, since fruit is the main component of a healthy daily diet. Its contribution lies in it having a set of beneficial nutritional and non-nutritional substances, such as vitamin C, carotenoids, flavonoids, phytosterols, among other bioactive compounds, able to provide antioxidant activity [1]. The activity of the antioxidant compounds has been related to the prevention of a wide range of pathologies, such as cancer, cardiovascular diseases and degenerative diseases, associated with aging processes [2]. However, the worldwide average intake of fruit is below that recommended by the health authorities. It is thought that the population does not consume fruit and vegetables for various reasons: cost, convenience and taste, among others [3].

For this reason, the food manufacturing sector has seen an opportunity to develop new healthy fruit-based products in order to encourage its consumption. In addition, as consumers are more aware of the benefits of healthy food, they are demanding more and more new healthy products, which, at the same time, must be innovative and ready-to-eat. In this sense, the commercialization of dried fruit-based products can offer two things: solutions to problems related to the short shelf life of fruits and their seasonality and a means of providing microbiologically stable products as a consequence of their low water activity. Furthermore, dried fruit both facilitates the shipping operations and makes them more profitable due to its lower volume and weight and easier handling. In addition, dehydration can be used to provide different food formats, such as a fruit snacks, or powdered fruit destined to be consumed rehydrated as a juice or infusion, or to be added to desserts, dairy products, salads, ice cream, among other things, and even for enriching almost any food in bioactive compounds [4,5].

Of the dehydration techniques, freeze-drying is one of the drying methods that provides the highest retention of chemical profile and antioxidant activity in foods, attributed to its less intense heating [6]. In fact, several studies have successfully obtained a vegetable/fruit snack with good physical, chemical and functional properties by using freeze-drying [7,8,9,10,11,12]. Despite its advantages, freeze-drying is one of the techniques with the highest energy consumption as it requires a long drying time [5,6,13]. If compared with another dehydration technique, such as spray-drying, known for being cheaper and leading to products of similar quality, Flink [13] reported that the costs of freeze-drying are 4–5 times higher. However, a recent study on the obtaining of grapefruit powder points out that it is much more profitable to use freeze-drying rather than spray-drying, the latter costing 2.3 times more than the former [14]. This study indicated that although the electrical cost was 8.5 times higher in the case of freeze-drying, the costs of other items that intervene in the total cost of production, such as the raw material, is 3.5 times higher in the case of spray-drying; this represents 82% of its total cost, compared to 54% for freeze-drying. This is mainly due to the poor yield of spray-drying in the case of fruit processing, caused by the loss of the powder that remains adhered to the walls and nozzle of the spray-dryer [14]. This is a consequence of the structural collapse that the powdered fruit suffers at temperatures above its glass transition temperature (Tg) which, among other things, implies the development of stickiness phenomena. In this sense, certain biopolymers that contribute to an increase in the Tg, stabilizing the dehydrated product [15], are frequently incorporated.

In relation to the high energy consumption involved in freeze-drying, it has been reported that modifying the process variables, such as shelf temperature or working pressure, may affect the duration of the process, and the lower the pressure and the higher the temperature, the lower the process time [5,16]. However, a key issue to consider is whether the shorter process time compensates for the increased energy consumption of lower pressure and higher temperature applied in order to try to reduce the product cost. On the other hand, as regards the drying temperature, its maximum value will be limited both to prevent ice melting while ice is present in the product and to prevent significant quality changes [5]. In this sense, Silva-Espinoza et al. [11] pointed out that, in general, a moderate variation in the above-mentioned process variables had a low impact on the physical-chemical quality and bioactive compounds of the final freeze-dried product. In fact, taking vitamin C as a reference of high nutritional quality for foods under different industrial processes, due to its relative instability to heat, oxygen and light [17], a whole preservation after freeze-drying was observed, even when applying mild shelf temperatures such as 40 or 50 °C [11,18]. Moreover, phenolic compound content, as well as total flavonoids, flavanols, flavones, catechins in freeze-dried samples, showed high stability after the freeze-drying process [5,19,20].

Taking all of the above into account, this study has evaluated the effect that modifying the drying conditions (working pressure and shelf temperature) of the frozen sample, in order to obtain a freeze-dried orange snack, had on total energy consumption. The final aim was to select a proper combination of these variables to allow us to achieve an appropriate time-saving, energy-saving, economical process so as to ensure a high quality product.

## 2. Materials and Methods

### 2.1. Raw Material and Formulation

The oranges (Citrus × sinensis cultivar Navelina) used in this study were purchased from a local supermarket in the city of Valencia (Spain). They were selected by means of a visual inspection based on a similar weight and size, colour homogeneity and good physical integrity. The oranges were processed on the day of purchase. The carriers used to obtain a stable dehydrated orange puree were gum Arabic (GA, Scharlab, Sentmenat, Spain) and bamboo fibre (BF, VITACEL^®^, Rosenberg, Germany). This mix of biopolymers was selected because, while being equally effective as others in protecting against changes in different physical properties, it was shown to be more effective for phytochemicals protection during the intestinal stage of digestion [18,19]. In order to obtain the orange puree, the fruit was washed and peeled. The pulp was cut and triturated in a bench top electrical food processor (Thermomix TM 21, Vorwerk, Madrid, Spain) and mixed with (5 g GA + 1 g BF)/100 g orange puree with the same food processor. The conditions of trituration and mixing employed by Silva-Espinoza et al. [11] were followed.

### 2.2. Freeze-Drying Conditions

The formulated orange puree was distributed in 10.5 cm × 7.8 cm aluminum plates with a sample thickness of 0.5 cm and immediately frozen. A 0.2 cm diameter PT100 probe (Termya, Madrid, Spain) was placed in the geometric centre of the sample, which was frozen in a conventional freezer (Liebherr Mediline LGT 2325, Liebherr, Baden-Wurtemberg, Germany) for 48 h. The drying step was carried out in a freeze-dryer (Telstar Lyo Quest-55, Telstar, Terrassa, Spain) at two different chamber pressures, 5 and 100 Pa (P5 and P100, respectively) and at three different shelf temperatures, 30, 40 and 50 °C. These conditions have been chosen because, in a previous study, it was found that they neither affect the physical properties nor the bioactive compound content of this product [11]. In this way, six different conditions were studied and the obtained samples were coded as 30_P5, 30_P100, 40_P5, 40_P100, 50_P5, 50_P100. In each case, the drying time was adjusted so that it was enough to obtain a freeze-dried product with the same water content, lower than 5%, which guarantees the typical crispness of a snack [21]. Based on preliminary experiments, this time was 11 h for 30_P5, 27 h for 30_P100, 7 h 20 min for 40_P5, 8 h 30 min for 40_P100, 5 h 50 min for 50_P5 and 6 h for 50_P100. The same amount of sample (about 160 g, 4 plates) was loaded into the freeze-dryer for each of the six process conditions.

### 2.3. Water Content

The water content of the freeze-dried samples was measured offline with an automatic Karl-Fischer titrator (Mettler Toledo, Compact Coulometric Titrator C10S, Worthington, OH, USA) based on the chemical reaction of iodine in the presence of water [22,23]. Three replicates were made for each sample.

### 2.4. Power Consumption and Temperature Record

The energy consumption during the drying step was recorded with a real time data logger (Lutron Electronic Enterprise Co., LTD, Taipey City, Taiwan) that was connected to the freeze-dryer. The power consumed (W) was registered every 15 s.

The evolution of the temperature of the sample during the drying step was recorded every 30 s using a wireless system consisting of a data transmitter (Datanet Logger DNL910A, Fourtec Technologies Ltd., Rosh Ha’ayin, Israel), coupled to the Pt 100 probe described in Section 2.2, and a data receiver (Datanet Repeater DNR900, Fourtec Technologies Ltd., Rosh Ha’ayin, Israel) connected to a computer using DataSuite 3.0 software (Fourtec Technologies Ltd., Rosh Ha’ayin, Israel).

### 2.5. Statistical Analysis

An analysis of variance (ANOVA) was performed in order to obtain the significant differences between the various studied conditions according to Tukey’s HSD test, which were considered when *p* < 0.05, using Statgraphics Centurion XVI.II [24].

## 3. Results and Discussion

**Figure 1:**
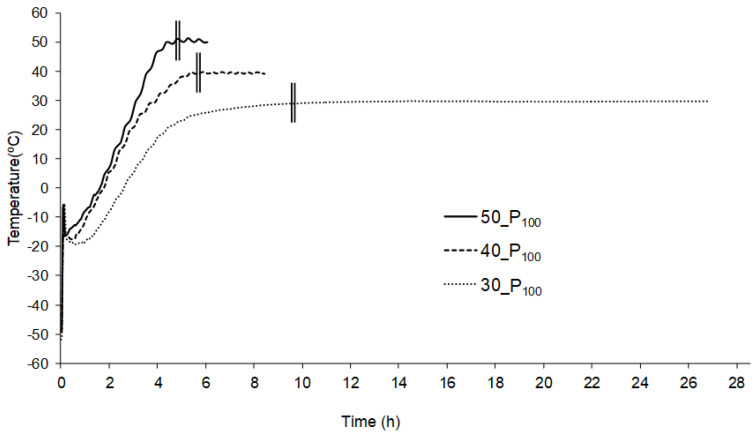
Evolution of the product temperature during the drying of the frozen orange puree at a working pressure of 100 Pa (P_100_) and a shelf temperature of 30, 40 or 50 °C A double vertical line indicates the end of primary drying and the beginning of secondary drying.

Table 1 shows the water content of the different orange snacks, all of which were crispy products with a water content of around 4% and no significant differences (*p* > 0.05). The temperature evolution of the orange snack in the different drying conditions was registered. Figure 1 shows the progress of the product temperature during the drying at 30, 40 and 50 °C. A similar trend was observed when varying the working pressure. The temperature evolution was similar to that obtained with other products [25] and both the primary and secondary drying stages can be observed [5,8,26,27]. The primary drying (PD) consists mainly of the transformation of the ice into vapour by sublimation, while the secondary drying (SD) refers to the desorption of the unfrozen water [5].

The precise end of PD is difficult to establish, as SD starts in some regions of the sample at the same time as primary drying ends in some others. If some of the non-frozen water is desorbed in the dried layer during the primary drying stage, the amount of heat that arrives at the sublimation interface may decrease and thus the rate of advance at the sublimation front [5]. Ratti [26] identified the start of SD as the moment when the product temperature begins to increase markedly. Other authors indicated that SD mostly occurs when the product temperature approaches the shelf temperature and so they assumed that point as the SD starting point [28,29,30,31]. In this study, we were able to observe that the samples dried at 100 Pa and 50 or 40 °C for 4 and 5 h, respectively, showed the presence of ice. As these times correspond to the moment when the product temperature truly approaches that of the shelf (Figure 1), in this study it was considered that the SD started when the product reached the shelf temperature.

From this point of view, as shown in Figure 1, the first period of PD is characterized by a low and constant product temperature where the sublimation is mainly taking place, using the heat provided by the shelf to supply the needed phase transition latent heat. In the second period of PD, in which less ice is left, less heat is needed for the sublimation and the product temperature rises. The duration of these two periods of PD was conditioned by the process variables (Figure 1). When comparing different shelf temperatures, the first period can be clearly observed to be shorter and a steeper slope of the product temperature increase can be seen in the second period when working at the higher temperatures (40 and 50 °C). It seems that the heat provided by the shelves when heated at these temperatures is more than the 2840 kJ/kg ice needed for the sublimation to occur [5], so that almost from the beginning, the temperature of the product begins to increase. On the other hand, during SD, the removal of the residual water content takes place until reaching the target value and the product heating rate greatly decreases until the shelf temperature is reached.

**Figure 2:**
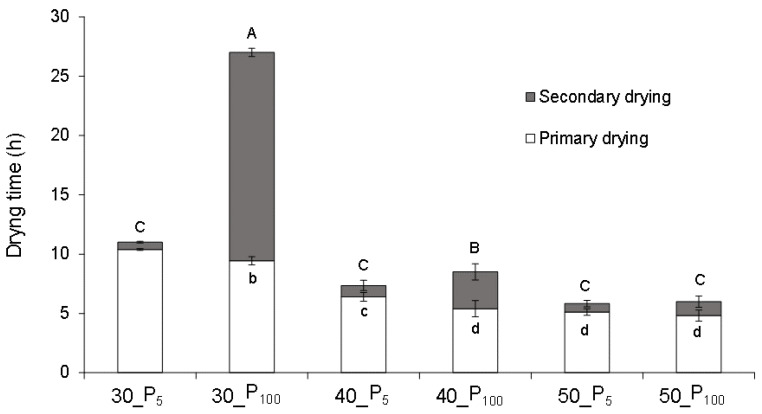
Mean value and standard deviation of the duration of primary and secondary drying for the frozen orange snacks dried at 5 Pa (P5) or 100 Pa (P100) and a shelf temperature of 30, 40 or 50 °C. Different lowercase or capital letters for primary drying and secondary drying, respectively indicate different homogeneous groups (*p* < 0.05) according to Tukey’s HSD test.

Taking into account the aforementioned criterion that the SD started when the product reached the shelf temperature, Figure 2 shows the duration of PD and SD for each of the process conditions studied. According to the ANOVA, both the shelf temperature and the pressure significantly affected (*p* < 0.05) the duration of PD and SD (Figure 2). Both a higher pressure and a higher temperature mean a shorter PD. In this study, this stage was significantly longer (*p* < 0.05) when the drying was done at 30 °C, at any pressure, compared to the other 2 temperatures studied. In the case of 40 °C, PD was significantly longer (*p* < 0.05) compared to 50 °C, when the former was carried out at the lowest pressure only. Ratti [26] explains that a higher pressure in the chamber enhances the heat transfer rate and, therefore, increases the sublimation rate, shortening the PD time. As regards SD, at P100 it was shorter as the shelf temperature increased, but this effect of temperature was not observed at P5 (*p* < 0.05, Figure 2). In addition, at 30 and 40 °C, the SD lengthened as the pressure increased. This effect of a higher pressure during the SD is related to the higher partial pressure of water vapour in the chamber [32] and the decrease in the diffusivity of water vapour in air [33], which delays the desorption of the unfrozen water.

As regards the duration of each drying step, it can be said that it depends on the freeze-drying conditions. Some authors indicated that primary drying normally consumes the largest fraction of the freeze-drying cycle time [34]. However, it has also been observed that the time required to remove the water during the secondary stage may be equivalent to, or even longer than, that in the primary stage [8,35]. In this study, SD was longer than PD only when the drying was carried out at 30 °C and P100 (Figure 2). Broadly speaking, when the drying was carried out at the lowest pressure, drying time reductions of 59%, 14% and 3% at 30, 40 and 50 °C, respectively, were observed. On the other hand, time reductions of 33% and 47% at P5 and 68% and 78% at P100 were achieved when drying at 40 and 50 °C, respectively, as compared with the process carried out at 30 °C.

**Figure 3:**
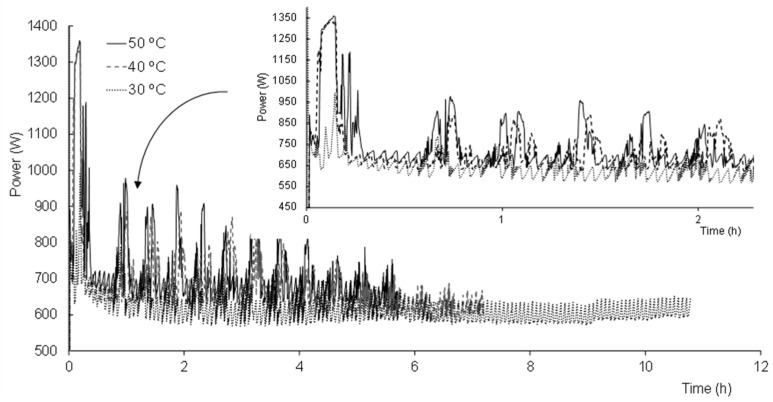
Evolution of the power consumed (W) over time (h) during the drying of the frozen orange puree at a working pressure of 5 Pa and a shelf temperature of 30, 40 and 50 °C.

**Figure 4:**
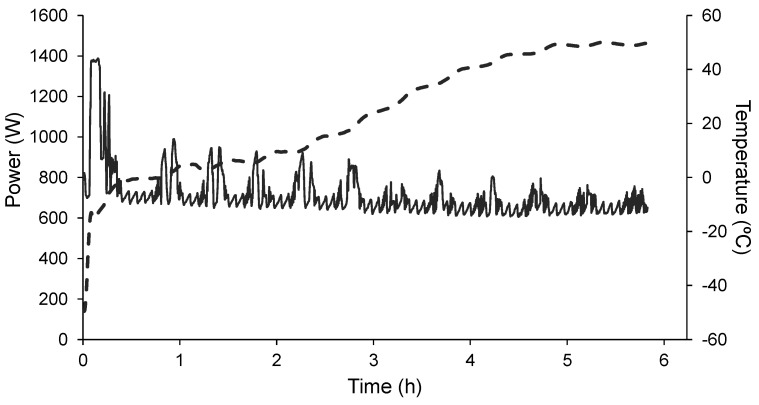
Evolution of the power consumed (continuous line) and of the product temperature (dashed line) over time (h) during the drying of the frozen orange puree at a working pressure of 5 Pa and a shelf temperature of 50 °C.

Figure 3 shows, as an example, the evolution of the power consumed throughout the drying step of three of the samples when applying the same pressure but different shelf temperatures, of 30, 40 and 50 °C. Each peak of power observed throughout the process is related to the operation mode of the freeze-dryer that has to supply heat for two purposes: so that the shelf temperature reaches and remains at the set value and in order to remove the water from the sample. As expected, a greater power consumption was observed at the beginning of the processes at 40 and 50 °C, which was necessary to reach the higher shelf temperature set point. On the other hand, more intense peaks are observed at any temperature, at the beginning of the primary drying, while the sublimation of the ice is clearly taking place (Figure 4). Since less ice is left as the sublimation progresses and the product temperature starts to rise, the shelves are allowed to preserve the temperature set point much better, so that the intensity of the power peaks decreases as the drying progresses (Figure 4).

The total power consumed by each of the different process conditions was obtained from the registered power consumption data, calculated from the enclosed area under the curve using the midpoint rectangular method (Table 1). Both shelf temperature and working pressure had a significant effect on the total power consumption (*p* < 0.05). On the one hand, although at no given moment was the required energy input higher when the drying was carried out at 30 °C, a greater amount of total power was consumed due to the much longer process (Table 1, *p* < 0.05). On the other hand, although significant, the difference in the energy consumption of the processes carried out at 40 or 50 °C was very small. This behaviour is related to the heat required for the drying of the product. Drying at 30 °C does not provide enough heat to remove the water content from the product in a reasonable time, which is achieved at 40 °C. From then on, 50 °C also allows the process to be shortened a little more, although it is not a great deal more effective. Nevertheless, taking into account that the vitamin C and β-carotene of the product was not observed to be affected by heating the shelves to 50 °C [11], this would be the recommended temperature with which to obtain the orange snack. A higher pressure increased the total power consumption (*p* < 0.05), except for the freeze-drying carried out at 50 °C (Table 1). This increase at P100 is also related to the longer duration of the process needed to achieve the target water content.

## 4. Conclusions

Even though heating the freeze-dryer shelves during the drying stage implies an increase in the energy consumed at specific moments, this allows for a shorter process time, so that less total power is consumed. A lower working pressure also permits a shorter drying time, leading to a significant decrease in energy consumption at lower temperatures. From this point of view, 50 °C and P5 may be recommended as the freeze-drying conditions with which to obtain a more economical orange snack.

## Figures and Tables

**Table 1:** Values of the water content of the orange snacks and total power consumed during the drying (mean ± standard deviation) according to each process condition. Different lowercase letters (a–e) in each column indicate different homogeneous groups (*p* < 0.05) according to Tukey’s HSD test.

Shelf Temperature	Pressure	Water Content (g Water/100 g Orange Snack)	Total Power Consumed (kWh)
30 °C	P_5_	3.7 ± 0.2 ^a^	6.83 ± 0.06 ^b^
	P_100_	3.8 ± 0.13 ^a^	16.49 ± 0.12 ^a^
40 °C	P_5_	3.6 ± 0.5 ^a^	5.03 ± 0.12 ^d^
	P_100_	3.8 ± 0.4 ^a^	5.72 ± 0.06 ^c^
50 °C	P_5_	3.6 ± 0.3 ^a^	4.14 ± 0.05 ^e^
	P_100_	3.80 ± 0.12 ^a^	4.23 ± 0.09 ^e^

## Data Availability

The data presented in this study are available on request from the corresponding author.
